# An open-label, flexible dose adaptive study evaluating the efficacy of vortioxetine in subjects with panic disorder

**DOI:** 10.1186/s12991-018-0190-6

**Published:** 2018-05-11

**Authors:** Anish Shah, Joanne Northcutt

**Affiliations:** 1 Siyan Clinical Corporation, 480 Tesconi Dr., Santa Rosa, CA 95401 USA; 2 Northcutt Consulting, LLC, Orlando, USA

**Keywords:** Panic disorder, Vortioxetine, Anxiety, Agoraphobia, Depression

## Abstract

**Background:**

Despite the current treatments available for panic disorder (PD), as many as one-third of patients have persistent and treatment-resistant panic attacks. Vortioxetine is an approved medicine for major depressive disorder and has been shown to have anxiolytic properties. The purpose of this study was to evaluate its efficacy and safety in an adult population with a diagnosis of PD.

**Methods:**

The study design was open label with flexible dose strategies (5, 10, or 20 mg) with a treatment period of 10 weeks. 27 male and female subjects aged between 18 and 60 years, who met DSM-IV criteria for PD with or without agoraphobia, or who had a Panic Disorder Severity Scale (PDSS) score > 8 at baseline were enrolled. Statistical significance was established by the Student’s *T* test.

**Results:**

A statistically significant decrease in the occurrence of panic attacks was measured with the PDSS with vortioxetine. In addition, a moderate improvement in the quality of life and no significant side effects were observed using the Quality-of-Life Scale and Monitoring of Side Effects Scale, respectively.

**Conclusions:**

These results provide some support for the use of vortioxetine in the management of panic disorder.

*Trial registration* ClinicalTrials.gov ID#: NCT02395510. Registered March 23, 2015, https://clinicaltrials.gov/ct2/show/NCT02395510

## Background

Panic disorder (PD) is a common anxiety disorder affecting up to 5% of the population at a given time [1, 2]. It is characterized by one or more unexpected panic attacks followed by anticipatory worry about additional attacks, including a morbid fear of death. PD has been strongly linked to age, female gender, smoking, and depleted financial resources [3]. It can be severely disabling and can greatly worsen quality of life when associated with agoraphobia [2, 4]. PD has a strong co-morbidity with other anxiety disorders, especially social phobia and obsessive–compulsive disorder [5].

There are multiple neurobiological theories posited with regard to the genesis of PD. Three chemicals seem to be of particular importance: serotonin (5-HT), noradrenaline, and gamma-aminobutyric acid (GABA) [6]. While there are also other neurobiological mechanisms involving corticotrophic releasing factor (CRF), dysfunction of the hypothalamic–pituitary–adrenal gland, and disrupted GABA and glutamate activity, the following are the proposed mechanisms of action controlling panic attacks:

1. Whereas 5-HT neurons located at the dorsal raphe nucleus are involved in the regulation of both inhibitory avoidance and escape, those of the median raphe nucleus are primarily implicated in panic attacks.
2. Facilitation of 5-HT1A- and 5-HT2A-mediated neurotransmission in the dorsal periaqueductal gray (dPAG) is likely to mediate a panicolytic drug action.
3. Stimulation of 5-HT2C receptors in the basolateral amygdala increases anxiety and is implicated in the anxiogenesis caused by short-term administration of antidepressant drugs.
4. 5-HT1A and the µ-opioid receptors work together in the dPAG to modulate escape or panic attacks.

In one meta-analysis of PD treatments, medications (especially serotonin–noradrenaline reuptake inhibitors) were found to be more effective based on calculated effect sizes, compared to psychotherapies and other therapies [7]. PD is generally treated very effectively with benzodiazepines, but these medications have a high risk for addiction, physical weakness, and cognitive impairment [8, 9] indicating the need for more effective long-term treatment. There are multiple drugs within the selective serotonin reuptake inhibitor (SSRI) and serotonin and norepinephrine reuptake inhibitor (SNRI) class that have proven efficacy in treating patients with PD [10–16]. Studies of treatment with paroxetine, venlafaxine, and escitalopram indicate positive results in the treatment of PD. The following antidepressants are significantly superior to placebo for PD patients, listed in increasing order of effectiveness: escitalopram, sertraline, paroxetine, fluoxetine, and venlafaxine for panic symptoms; paroxetine, fluoxetine, fluvoxamine, venlafaxine, and mirtazapine for overall anxiety symptoms [10–16].

Vortioxetine is an atypical antidepressant that has been approved for the treatment of the major depressive disorder [17]. The primary aim of this study was to evaluate the efficacy of vortioxetine in an adult patient population of PD with or without agoraphobia. Although the unique, multimodal mechanism of action of vortioxetine is not fully understood, it is thought to be related to enhancement of serotonergic activity in the CNS through inhibition of the reuptake of serotonin (5-HT) [18]. It also has several other functions, including 5-HT3 receptor antagonism and 5-HT1A receptor agonism. In vitro studies have shown it to be a 5HT1D and 5HT7 receptor antagonist and 5HT1B receptor partial agonist [19, 20].

In mouse models of anxiety and depression-like behavior, the antidepressant and anxiolytic effects produced by vortioxetine were greater than those produced by fluoxetine and comparable (in the open-field test) with those produced by diazepam [21]. In humans, a double-blind, randomized, fixed-dose (15 or 20 mg), placebo-controlled study showed that scores on the Hamilton Anxiety Rating Scale (HAM-A) decreased significantly from baseline with regular doses of vortioxetine [22]. No statistically significant gender differences have been observed in its action [23].

Based upon the evidence for the multimodal action of the drug and recent published data, the authors believe that vortioxetine may have clinical relevance in the treatment of PD, and our primary aim was to provide support for this hypothesis. The secondary aim of this study was to monitor the quality of life of subjects with PD when treated with vortioxetine.

## Methods

### Study design and patient recruitment

**Fig. 1:**
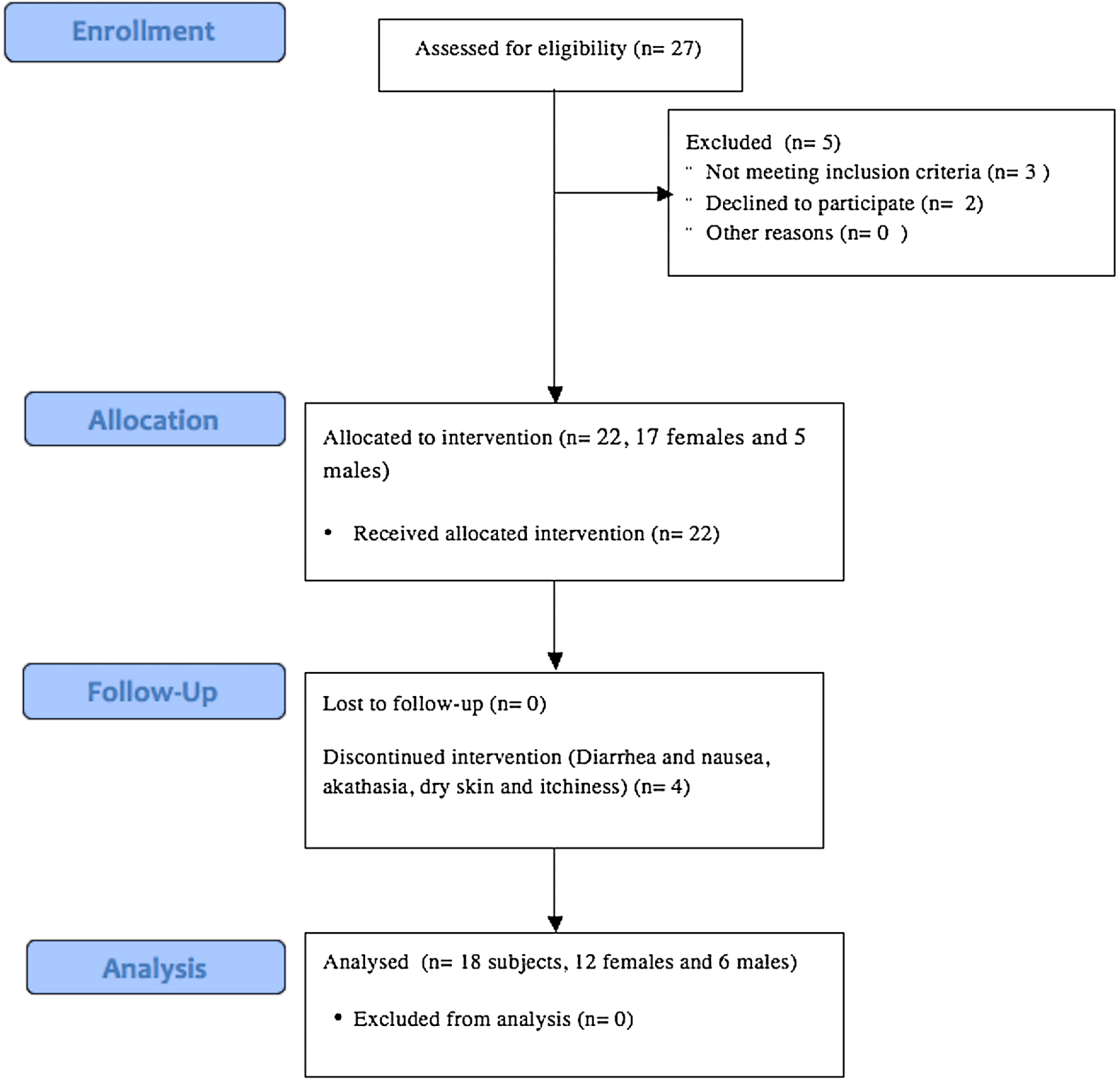
TREND flowchart for patient recruitment, screening, analysis and follow-up

We designed an open-label 10 week-long study with fixed doses (5, 10, or 20 mg) to evaluate the safety, tolerability, and efficacy of vortioxetine for the treatment of PD, see Fig. 1 for the transparent reporting of evaluations with nonrandomized design (TREND) flowchart. 27 male and female subjects aged between 18 and 60 years, who met DSM-IV criteria for PD with or without agoraphobia, or who had a Panic Disorder Severity Scale (PDSS) score > 8 at baseline were screened. Institutional Review Board (IRB) approval for this study was obtained from the Schulman Associates IRB (Approval #: 201500056) and this study was conducted according to the World Medical Association Declaration of Helsinki. Only patients with active ongoing panic disorder were selected (as diagnosed by the Investigator as per the Diagnostic and Statistical Manual of Mental Disorders-Fourth Edition [DSM-IV] criteria using the MINI International Neuropsychiatric Interview 6.0); the previous treatment had not succeeded in eliminating their symptoms. Five patients were excluded for not meeting inclusion criteria, or not wishing to participate. After providing written informed consent, 22 subjects underwent a 7–14 day washout period of their current SSRI or SNRI medications. In the case of a subject taking a medication with a long half-life such as fluoxetine, a 5-week washout period prior to Baseline was allowed. There was no washout requirement for subjects using benzodiazepines or hypnotics prior to study enrollment. Subjects were allowed to continue concomitant treatment with these medications during the study; however, treatment was not initiated with benzodiazepines or hypnotics for any of the enrolled subjects, which allowed for a better overall assessment in the efficacy of vortioxetine for panic disorder. All subjects who participated in the study met the designated inclusion criteria and none of the exclusion criteria. Four patients discontinued the study, suffering from diarrhea, nausea, akathisia, dry skin, and itchiness. All patients with adverse events were monitored, serious, or otherwise. One patient suffered a serious adverse event (SAE) of a pulmonary embolus after completing the study; this event was not deemed as related to the study treatment.

### Dosing

Subjects received 5 mg of vortioxetine and were instructed to take one capsule orally, once daily in the morning, starting at the baseline visit (visit 1). Subjects were then seen bi-weekly following the baseline visit for a period of 10 weeks. The study medication was given on a flexible titration schedule until an optimal dose or response (in the opinion of the investigator) had been achieved for each subject. This design allowed the investigator to focus on the most effective dose for each subject with the total daily dose of vortioxetine not exceeding 20 mg.

### Assessments

Two efficacy and two safety assessments were used. The efficacy assessments consisted of the Panic Disorder Severity Scale (PDSS) and the Quality-of-Life Scale (QOLS). The PDSS is a questionnaire developed for measuring the severity of PD and is considered a reliable tool for monitoring treatment outcome [24, 25]. The safety assessments included the Monitoring of Side Effects Scale (MOSES) and the Columbia-Suicide Severity Rating Scale (C-SSRS) [26, 27].

### Statistical analysis

*T* tests were applied to compare the baseline scores for PDSS, QOLS, and MOSES to those for the same at the completion of the study for 18 patients (12 females and six males). Analyses and descriptive statistics were obtained using the R 3.1.1 statistical software (R foundation for Statistical Computing, Vienna, Austria).

## Results

### Patient population characteristics

**Fig. 2:**
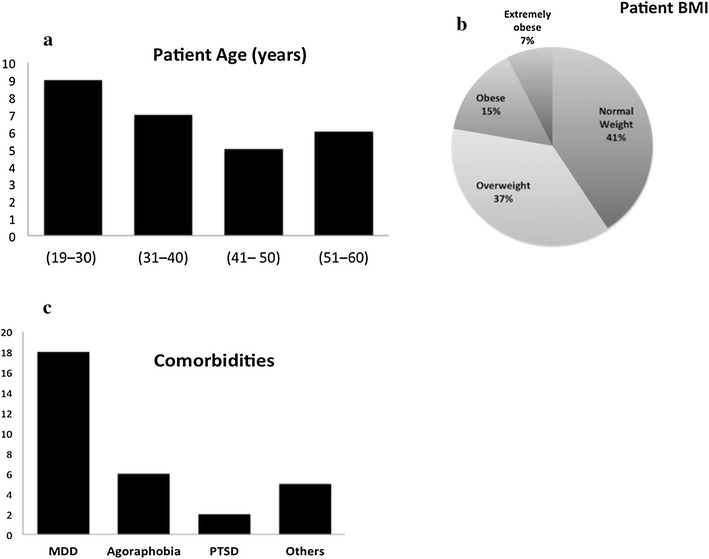
**a** Patient age distribution. **b** Patient BMI distribution. **c** Existing comorbidities in the patient population

From March to May 2016, 27 subjects were screened in the study, of which 20 were female. The median [IQR] age of the subjects was 39 (age range 23.5–49 years) (Fig. 2a). The median BMI of the patient population was 26.25 (range 23.58–29.2), and 59% were overweight (Fig. 2b). Major depressive disorder was the most frequent co-morbidity in this patient population, followed by agoraphobia (Fig. 2c).

### Significant decrease observed in overall PDSS scores and the frequency of panic attacks

**Fig. 3:**
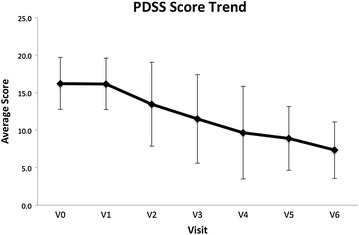
Graph showing PDSS score trend over the course of the study, a statistically significant decrease was observed in the PDSS scores upon completion of the study (*p* < 0.05)

PDSS score was used as a reliable method for measuring the severity of PD on each clinic visit. PDSS scores assess seven important criteria: panic frequency, distress during the panic attack, panic-focused anticipatory anxiety, phobic avoidance of situations, phobic avoidance of physical sensations, impairment in work functioning, and impairment in social functioning, and provide an overall score for PD severity. A total of 18 subjects, 12 females and six males, completed the study. A statistically significant decrease was observed between the baseline PDSS scores and final scores upon completing the study (*p* < 0.05), with a mean decrease of 8.89 (Fig. 3).

With respect to PDSS question one, regarding the frequency of panic attacks, a statistically significant improvement was observed between the baseline scores and those at the completion of the study (*p* < 0.05), with an observed decrease of 1.11 in the mean score.

### Statistically significant decrease observed in the MOSES score

**Fig. 4:**
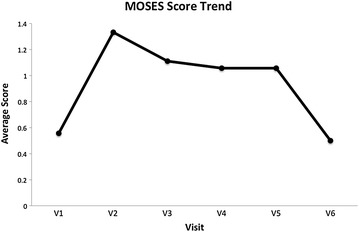
Graph showing monitoring of the side effects over the course of the study, a significant decrease was observed in the MOSES score (*p* = 0.014)

The MOSES scale was used to document the safety and tolerability of vortioxetine. The MOSES has 73 items presented in layperson’s language organized into nine body areas representing a typical physical examination. A statistically significant decrease (an improvement) was observed between the baseline MOSES score and the score upon completing the study (*p* = 0.014), with a mean decrease of 1.06 on the MOSES scale (Fig. 4).

### An increase observed in the QOL score

**Fig. 5:**
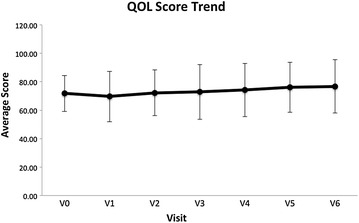
Graph showing quality-of-life scores over the course of the study, an increasing trend in the QOL score was observed upon completion of the study (*p* < 0.1 but > 0.05, *p* = 0.061)

The Quality-of-Life (QOL) scale is a valid instrument for measuring the quality of life across the patient groups [9]. The QOLS has 16 items rated on 7-point response scales. Higher scores indicate a higher quality of life. A trend towards an increase was observed in QOL score upon the completion of the study (*p* = 0.061), with a mean gain of 4.944 (Fig. 5).

### Limitations

The sample size is small (*n* = 22) and mostly consisted of female subjects; with 18 subjects completing the study. There was neither a placebo or active control arm, nor randomization of subjects used for this study.

## Discussion

Despite the current treatments available, as many as one-third of patients with PD have persistent and treatment-resistant panic attacks [28]. A previous meta-analysis study has shown that vortioxetine was efficacious in reducing depressive and anxiety symptoms in patients with MDD and high levels of anxiety [29]. We aimed to evaluate the effectiveness of vortioxetine in patients with panic disorder. Previous studies of vortioxetine efficacy have focused on different scoring measures, most notably the Hamilton Anxiety Rating Scale [30] and the personality diagnostic questionnaire (PDQ), which provides self-measurement of cognitive function [31]. We adopted the Panic Disorder Severity Scale which is a widely accepted clinical tool used for the measurement of panic disorder symptom severity. It has been shown to have acceptable internal consistency (Cronbach’s *α* = 0.65) and high inter-relater reliability with correlation coefficients ranging from 0.87 to 0.88 [32].

Using the PDSS, a statistically significant decrease in the occurrence of panic attacks was measured from baseline to study completion. The mean decrease in PDSS scores was 8.89 (*p* < 0.05), indicative of less severe PD. Regarding the frequency of panic attacks or question one on the scale, a statistically significant improvement was observed between baseline and study completion (*p* < 0.05), with an observed decrease of 1.11 in the mean frequency of attacks.

Administration of the MOSES scale provided an insight into the safety and tolerability of vortioxetine. The results of this monitoring showed a statistically significant decrease in the MOSES score upon study completion (*p* = 0.014), indicating a decrease in observed side effects. Likewise, the Quality-of-Life Scale (QOLS) showed an improvement in scores. This scale has been validated for measuring the quality of life across patient groups [8].

The prevalence of panic disorder remains high, with a large percentage of patients unable to remain gainfully employed and presenting with a higher rate of hospitalizations. Our results provide support for the use of vortioxetine in the management of panic disorder.

## Conclusions

Our study provides support for the use of vortioxetine in the management of panic disorder.

## Abbreviations

dPAG: dorsal periaqueductal gray
CRF: corticotrophic releasing factor
GABA: gamma-aminobutyric acid
MDD: major depressive disorder
MOSES: Monitoring of Side Effects Scale
PD: panic disorder
PDQ: personality diagnostic questionnaire
PDSS: Panic Disorder Severity Scale
QOLS: Quality-of-Life Scale
SNRI: serotonin and norepinephrine reuptake inhibitor
SSRI: selective serotonin reuptake inhibitor

## References

[1] Kessler RC, Chiu WT, Jin R, Ruscio AM, Shear K, Walters EE (2006). The epidemiology of panic attacks, panic disorder, and agoraphobia in the national comorbidity survey replication. Arch Gen Psychiatry.

[2] Roy-Byrne PP, Craske MG, Stein MB (2006). Panic disorder. Lancet.

[3] Moreno-Peral P, Conejo-Ceron S, Motrico E, Rodriguez-Morejon A, Fernandez A, Garcia-Campayo J, Roca M, Serrano-Blanco A, Rubio-Valera M, Bellon JA (2014). Risk factors for the onset of panic and generalised anxiety disorders in the general adult population: a systematic review of cohort studies. J Affect Disord.

[4] Taylor CB (2006). Panic disorder. BMJ.

[5] Camuri G, Oldani L, Dell’Osso B, Benatti B, Lietti L, Palazzo C, Altamura AC (2014). Prevalence and disability of comorbid social phobia and obsessive-compulsive disorder in patients with panic disorder and generalized anxiety disorder. Int J Psychiatry Clin Pract.

[6] Zwanger P, Rupprecht R (2005). Selective GABAergic treatment for panic? Investigations in experimental panic induction and panic disorder. J Psychiatry Neurosci.

[7] Bandelow B, Reitt M, Röver C (2015). Efficacy of treatments for anxiety disorders: a meta-analysis. Int Clin Psychopharmacol.

[8] Offidani E, Guidi J, Tomba E, Fava GA (2013). Efficacy and tolerability of benzodiazepines versus antidepressants in anxiety disorders: a systematic review and meta-analysis. Psychother Psychosom.

[9] Perna G, Alciati A, Riva A, Micieli W, Caldirola D (2016). Long-term pharmacological treatments of anxiety disorders: an updated systematic review. Curr Psychiatry Rep.

[10] Katzman MA, Jacobs L (2007). Venlaxifine in the treatment of panic disorder. Neuropsychiatr Dis Treat.

[11] Simon NM, Otto MW, Worthington LL (2009). Next-step strategies for panic disorder refractory to initial pharmacotherapy. J Clin Psychiatry.

[12] Fochtmann LJ (2000). Practice guideline for the treatment of patients with panic disorder.

[13] Perna G, Dacco S, Menotti R (2011). Antianxiety medication for the treatment of complex agoraphobia: pharmacological interventions for a behavioral condition. Neuropsychiatr Dis Treat.

[14] Pecknold JC, Luthe L, Iny L (1995). Fluoxetine in panic disorder: pharmacologic and tritiated platelet imipramine and paroxetine binding study. J Psychiatry Neurosci.

[15] Bakker A, van Balkom AJLM, Stein DJ (2005). Evidence-based pharmacotherapy of panic disorder. Int J Neuropsychopharmcol.

[16] Holt RL, Lydiard RB (2007). Management of treatment-resistant panic disorder. Psychiatry.

[17] Connolly KR, Thase ME (2016). Vortioxetine: a new treatment for major depressive disorder. Expert Opin Pharmacother.

[18] Katona CL, Katona CP (2014). New generation multi-modal antidepressants: focus on vortioxetine for major depressive disorder. Neuropsychiatr Dis Treat.

[19] Orsolini L, Tomasetti C, Valchera A (2016). New advances in the treatment of generalized anxiety disorder: the multimodal antidepressant vortioxetine. Expert Rev Neurother.

[20] Stahl SM (2015). Modes and nodes explain the mechanism of action of vortioxetine, a multimodal agent (MMA): enhancing serotonin release by combining serotonin (5HT) transporter inhibition with actions at 5HT receptors (5HT1A, 5HT1B, 5HT1D, 5HT7 receptors). CNS Spectr.

[21] Guilloux JP, Mendez-David I, Pehrson A, Guiard BP, Reperant C, Orvoen S, Gardier AM, Hen R, Ebert B, Miller S (2013). Antidepressant and anxiolytic potential of the multimodal antidepressant vortioxetine (Lu AA21004) assessed by behavioural and neurogenesis outcomes in mice. Neuropharmacology.

[22] Boulenger JP, Loft H, Olsen CK (2014). Efficacy and safety of vortioxetine (Lu AA21004), 15 and 20 mg/day: a randomized, double-blind, placebo-controlled, duloxetine-referenced study in the acute treatment of adult patients with major depressive disorder. Int Clin Psychopharmacol.

[23] Areberg J, Søgaard B, Højer AM (2012). The clinical pharmacokinetics of Lu AA21004 and its major metabolite in healthy young volunteers. Basic Clin Pharmacol Toxicol.

[24] Shear MK, Brown TA, Barlow DH, Money R, Sholomskas DE, Woods SW, Gorman JM, Papp LA (1997). Multicenter collaborative panic disorder severity scale. Am J Psychiatry.

[25] Shear MK, Rucci P, Williams J, Frank E, Grochocinski V, Vander Bilt J, Houck P, Wang T (2001). Reliability and validity of the Panic Disorder Severity Scale: replication and extension. J Psychiatr Res.

[26] Kalachnik JE (1999). Measuring side effects of psychopharmacologic medication in individuals with mental retardation and developmental disabilities. Ment Retard Dev Disabil Res Rev.

[27] Posner K, Melvin GA, Stanley B, Oquendo MA, Gould M (2007). Factors in the assessment of suicidality in youth. CNS Spectr.

[28] Friere RC, Zugliani MM, Garcia RF (2016). Treatment-resistant panic disorder: a systematic review. Expert Opin Pharmocother.

[29] Baldwin DS (2016). A meta-analysis of the efficacy of vortioxetine in patients with major depressive disorder (MDD) and high levels of anxiety. J Affect Disord.

[30] Bidzan L, Mahableshwarkar AR, Jacobsen P, Yan M, Sheehan DV (2012). Vortioxetine (Lu AA21004) in generalized anxiety disorder: results of an 8-week, multinational, randomized, double-blind, placebo-controlled clinical trial. Eur Neuropsychopharmacol.

[31] Mahableshwarkar AR, Zajecka J, Jacobson W, Chen Y, Keefe RS (2015). A randomized, placebo-controlled, active-reference, double-blind, flexible-dose study of the efficacy of vortioxetine on cognitive function in major depressive disorder. Neuropsychopharmacology.

[32] Keough ME, Porter E, Kredlow MA, Worthington JJ, Hoge EA, Pollack MH, Shear MK, Simon NM (2012). Anchoring the panic disorder severity scale. Assessment.

